# Correction: Synergistic antibacterial effects of colistin in combination with aminoglycoside, carbapenems, cephalosporins, fluoroquinolones, tetracyclines, fosfomycin, and piperacillin on multidrug resistant *Klebsiella pneumoniae* isolates

**DOI:** 10.1371/journal.pone.0251994

**Published:** 2021-05-14

**Authors:** The PLOS ONE Staff

The second author’s first and last name are incorrectly switched. The correct name is Ozioma F. Nwabor. The correct citation is: Ontong JC, Nwabor OF, Voravuthikunchai SP, Chusri S (2021) Synergistic antibacterial effects of colistin in combination with aminoglycoside, carbapenems, cephalosporins, fluoroquinolones, tetracyclines, fosfomycin, and piperacillin on multidrug resistant *Klebsiella pneumoniae* isolates. *PLOS ONE* 16(1): e0244673. https://doi.org/10.1371/journal.pone.0244673

In Table 3, the column headings are formatted incorrectly. Please see the correct Table 3 here. The publisher apologizes for the error.

**Table 3:** Minimum inhibitory concentrations of colistin resistant K. pneumoniae isolates against conventional antibiotics.

Isolates	Antibiotics (µg/mL)																
		Aminoglycosides			Carbapenem		Cephalosporins				Fluoroquinolones			Tetracycline		Fosfomycin	Penicillin
	COL	AMI	GEN	TOB	IMI	MER	CEFZ	CEFX	CEFD	CEFT	CIP	LEV	MOX	MIN	TIG	FOS	PIP
1NT4Ng/1	256	16	128	32	256	256	>1024	>1024	>1024	>1024	>256	512	512	128	8	>1024	>1024
1NT6Ng/1	256	16	128	32	256	256	>1024	>1024	>1024	>1024	>256	512	512	128	8	>1024	>1024
1NT6Tu/1	256	8	256	64	128	256	>1024	>1024	>1024	>1024	>256	512	512	128	8	>1024	>1024
1NT6R	256	16	128	64	128	256	>1024	>1024	>1024	>1024	>256	512	512	128	8	>1024	>1024
1NT7R	256	8	128	32	256	256	>1024	>1024	>1024	>1024	>256	512	512	128	8	>1024	>1024
1NT8Th	256	16	64	32	128	128	>1024	>1024	>1024	>1024	>256	512	512	128	8	>1024	>1024
1NT6Ng (CCU)/1	512	16	128	16	128	256	>1024	>1024	>1024	>1024	128	64	32	32	8	128	>1024
1NT6Th(CCU)/1	256	16	128	16	128	128	>1024	>1024	>1024	>1024	128	64	32	32	8	128	>1024
1NT6R(CCU)	256	16	128	32	128	128	>1024	>1024	>1024	>1024	256	512	512	128	8	>1024	>1024
1SK5R	>1024	4	4	8	128	256	4	512	256	4	32	0.50	4	16	16	32	16
1TR5R	>1024	4	0.50	1	32	32	2	4	0.25	1	4	0.25	4	0.25	8	0.5	0.5

AMI, Amikacin; CCU, Cardiac Care Unit; CEFD, Ceftazidime; CEFT, Ceftozane and Tazobactam; CEFX, Cefotaxime; CEFZ, Cefoperazone; CIP, Ciprofloxacin; COL, Colistin; FOS, Fosfomycin; GEN, Gentamicin; IMI, Imipenem; LEV, Levofloxacin; MER, Meropenem; MIN, Minocycline; MOX, Moxifloxacin; PIP, Piperacillin; TIG, Tigecycline; TOB, Tobramycin.

## References

[1] Ontong JC, Nwabor OF, Voravuthikunchai SP, Chusri S (2021) Synergistic antibacterial effects of colistin in combination with aminoglycoside, carbapenems, cephalosporins, fluoroquinolones, tetracyclines, fosfomycin, and piperacillin on multidrug resistant Klebsiella pneumoniae isolates. *PLOS ONE* 16(1): e0244673. 10.1371/journal.pone.0244673 33406110 PMC7787437

