# Supplementary material for: Synergistic antibacterial effects of colistin in combination with aminoglycoside, carbapenems, cephalosporins, fluoroquinolones, tetracyclines, fosfomycin, and piperacillin on multidrug resistant Klebsiella pneumoniae isolates
Source: PLoS One. 2021 Jan 6;16(1):e0244673. doi: 10.1371/journal.pone.0244673 (PMC7787437; doi:10.1371/journal.pone.0244673)
Supplement: S1 Table — (DOCX) [file pone.0244673.s001.docx]

**S1 Table.** **Demographic data, clinical characteristics, and outcomes of the patients with colonization due to colistin-resistant and carbapenem-resistant *K. pneumoniae***

| Patient | Hospital | Sex | Age (years) | Site of colonization | Comorbidities | Emergent condition for admission | Condition for admission | Initial ICU  Admission | Initial APACHE II score | Retention of medical device(s) | Previous use of antibiotic(s) | Day of ICU admission before obtaining specimens | Infection after obtaining specimens | Discharge Status |
| --- | --- | --- | --- | --- | --- | --- | --- | --- | --- | --- | --- | --- | --- | --- |
| 1 | Narathiwat | M | 45 | GC | DM, HTN, DLD | Yes | CAP | Yes | 21 | MV, UC, CVC | LVX, IPM | 9 | VAP due to CRAB | Survived |
| 2 | Narathiwat | M | 82 | GC | DM, CAD | Yes | CHF | No | 15 | MV, UC | CRO, MEM | 4 | VAP due to CRE (KP) | Dead |
| 3 | Narathiwat | F | 94 | GC | DM, CVD, CAD | Yes | Aspiration pneumonia | No | 12 | MV, UC | TZP, MEM | 5 | None | Dead |
| 4 | Narathiwat | M | 71 | Throat | DM, HTN, CAD | Yes | CHF | Yes | 20 | MV, UC, CVC | CRO, TZP, IPM | 4 | VAP due to CRAB | Dead |
| 5 | Narathiwat | M | 25 | Throat | None | Yes | Severe head injury | Yes | 18 | MV, UC | CRO, MEM | 3 | VAP due to CRPA | Survived |
| 6 | Narathiwat | F | 39 | Rectum | None | Yes | Severe blunt chest injury | Yes | 19 | MV, UC | TZP, IPM | 3 | None | Survived |
| 7 | Narathiwat | M | 49 | Rectum | DM, DLD, CKD | Yes | CHF | Yes | 21 | MV, UC, CVC | CRO, ETP | 6 | UTI due to CRE (EC) | Dead |
| 8 | Narathiwat | M | 52 | Rectum | COPD, CAD | Yes | COPD exacerbation | No | 17 | MV, UC | CRO, MEM | 5 | VAP due to CRAB | Dead |
| 9 | Narathiwat | F | 64 | ETT | HTN, CVD, CKD | Yes | Aspiration pneumonia | No | 15 | MV, UA | CRO, CLI, IPM | 5 | None | Survived |
| 10 | Songkhla | M | 65 | Rectum | COPD, CKD | Yes | CHF | Yes | 21 | MV, UC, CVC | CRO, ETP | 7 | VAP due to CRAB | Survived |
| 11 | Trang | M | 70 | Rectum | DM, CAD, CKD | Yes | CAP | Yes | 22 | MV, UC, CVC | LVX, MEM | 6 | UTI due to CRE (KP) | Dead |

GC; Gastric content, ETT; Endotracheal tube, DM; diabetes mellitus, HTN; hypertension, DLD; dyslipidemia, CAD; coronary artery disease, CVD; cerebrovascular disease, CKD; chronic kidney disease, COPD; chronic obstructive pulmonary disease, CAP; community-acquired pneumonia, CHF; congestive heart failure, MV; mechanical ventilator, UC; urinary catheterization, CVC; central venous catheterization, LVX; levofloxacin, IPM; imipenem, CRO; ceftriaxone, MEM; meropenem, TZP; piperacillin-tazobactam; ETP; ertrapenem, CLI; clindamycin, VAP; ventilator-association pneumonia, UTI; urinary tract infection, CRAB; carbapenem-resistant *A. baumannii*, CRE; carbapenem-resistant *Enterobacteriaceae*, KP; *K. pneumoniae* , CRPA; carbapenem-resistant *P. aeruginosa* , EC; *E.coli*.
