# Supplementary material for: Synergistic antibacterial effects of colistin in combination with aminoglycoside, carbapenems, cephalosporins, fluoroquinolones, tetracyclines, fosfomycin, and piperacillin on multidrug resistant Klebsiella pneumoniae isolates
Source: PLoS One. 2021 Jan 6;16(1):e0244673. doi: 10.1371/journal.pone.0244673 (PMC7787437; doi:10.1371/journal.pone.0244673)
Supplement: S2 Table — (DOCX) [file pone.0244673.s002.docx]

**S2 Table**. Minimum inhibitory concentration (MIC) and minimum bactericidal concentration (MBC) of colistin against *Klebsiella pneumoniae* clinical isolates.

Clinical isolates (n=85) Sample type MIC/MBC of colistin (µg/ml) **Hatyai hospital**

1HY1Ng/1 Nasopharynx 2/2

1HY1Th/1 Throat 2/4

1HY2Th/1 Throat 2/2

1HY2R/1 Rectal 2/4

1HY3Th Endotracheal tube 1/1

1HY3R Rectal 1/1

1HY4R/1 Rectal 1/4

1HY5Ng/1 Nasopharynx 2/2

1HY5Th/1 Throat 1/2

1HY8Ng/1 Nasopharynx 2/2

1HY9Tu Endotracheal tube 2/2

1HY9R/1 Rectal 2/4

1HY11Ng/1 Nasopharynx 1/2

1HY11Tu/1 Endotracheal tube 1/1

1HY11Th Throat 1/2

1HY11R/1 Rectal 1/1

1HY14Tu/1 Endotracheal tube 1/1

1HY14Th Throat 1/1

1HY15Th Throat 1/1

1HY15R Rectal 1/2

**Narathiwat hospital**

1NT4Ng/1 Nasopharynx 256/512

1NT6Ng/1 Nasopharynx 256/512

1NT6Tu/1 Endotracheal tube 256/>1024

1NT6R Rectal 256/>1024

1NT7R Rectal 256/1024

1NT8Th Throat 256/512

1NT6Ng (CCU)/1 Nasopharynx 512/>1024

1NT6Th (CCU)/1 Throat 256/>1024

1NT6R (CCU) Rectal 256/512

**Pattani hospital**

1PA2R/1 Rectal 2/2

1PA3Ng Nasopharynx 2/2

1PA3Th/1 Throat 2/2

1PA6Ng Nasopharynx 1/2

1PA6Th Throat 1/2

1PA7Th/1 Throat 1/1

1PA7R Rectal 1/2

1PA9R Rectal 2/2

1PA11R Rectal 1/1

1PA13Tu/1 Endotracheal tube 1/2

1PA18Ng/1 Nasopharynx 2/2

1PA18R Rectal 2/2

1PA19Ng/1 Nasopharynx 2/2

1PA19Tu/1 Endotracheal tube 2/2

1PA19R Rectal 1/4

1PA20Ng/1 Nasopharynx 2/2

1PA20Tu/1 Endotracheal tube 2/2 1PA20Th/1 Throat 2/4

1PA20R Rectal 2/2

1PA20E Environment 2/2

1PA22Th/1 Throat 1/2

1PA22R Rectal 2/2

**Phatthalung hospital**

1PT2Ng Nasopharynx 1/2

1PT2Th/1 Throat 2/2

1PT2R/1 Rectal 2/2

1PT5Th/1 Throat 2/2

1PT5R/2 Rectal 2/4

1PT7Th/1 Throat 1/2

1PT7E/1 Environment 1/2

1PT10Th/1 Throat 2/2

1PT11Ng Nasopharynx 1/1

1PT11Tu/1 Endotracheal tube 1/1

1PT11Th/1 Throat 1/2

1PT11R Rectal 1/2

1PT11E/1 Environment 1/2

1PT12R Rectal 1/2

1PT13Ng Nasopharynx 1/1

1PT14Ng/1 Nasopharynx 2/2

**Songklanagarind hospital**

1PSU1R/1 Rectal 1/2

1PSU2R/1 Rectal 1/2

1PSU2R/2 Rectal 1/2

1PSU3Th Throat 0.5/2

1PSU3R Rectal 2/4

1PSU4Ng Nasopharynx 1/2

1PSU6Ng/1 Nasopharynx 1/2

**Songkhla hospital**

1SK1Ng/1 Nasopharynx 1/2

1SK5R Rectal >1024

1SK8Ng/1 Nasopharynx 2/2

**Satun hospital**

1ST3Ng Nasopharynx 1/1

1ST3R/1 Rectal 2/4

1ST5Th Throat 2/4

1ST9Ng/1 Nasopharynx 2/4

1ST9R/1 Rectal 2/2

1ST9R/2 Rectal 2/2

1ST13R Rectal 1/2

**Trang hospital**

1TR5R Rectal >1024

**Reference standard**

*Escherichia coli* ATCC 25922 0.25/0.5
