# Supplementary material for: Synergistic antibacterial effects of colistin in combination with aminoglycoside, carbapenems, cephalosporins, fluoroquinolones, tetracyclines, fosfomycin, and piperacillin on multidrug resistant Klebsiella pneumoniae isolates
Source: PLoS One. 2021 Jan 6;16(1):e0244673. doi: 10.1371/journal.pone.0244673 (PMC7787437; doi:10.1371/journal.pone.0244673)
Supplement: S3 Table — (DOCX) [file pone.0244673.s003.docx]

**S3 Table.** Synergistic effects of antibiotics and colistin combination against *Klebsiella pneumoniae* isolates from hospitalized patients.

Clinical isolates MIC (μg/ml) FICI Outcome

Combination Colistin Meropenem Colistin Meropenem

1NT4Ng/1 256 256 64 64 0.500 Synergistic

64 32 0.375 Synergistic

1NT6Ng/1 256 256 64 64 0.500 Synergistic

64 32 0.375 Synergistic

1NT6Tu/1 256 256 64 64 0.500 Synergistic

32 64 0.375 Synergistic

16 64 0.312 Synergistic

1NT6R 256 256 64 64 0.500 Synergistic

64 32 0.375 Synergistic

32 64 0.375 Synergistic

16 64 0.312 Synergistic

1NT7R 256 256 64 64 0.500 Synergistic

64 32 0.375 Synergistic

64 16 0.312 Synergistic

64 8 0.281 Synergistic

32 64 0.375 Synergistic

32 32 0.250 Synergistic

16 64 0.312 Synergistic

1NT8Th 256 128 64 32 0.500 Synergistic

1NT6Ng (CCU)/1 512 256 128 64 0.500 Synergistic

64 64 0.375 Synergistic

32 64 0.312 Synergistic

1NT6Th (CCU)/1 256 128 64 32 0.500 Synergistic

1NT6R (CCU) 256 128 64 32 0.500 Synergistic

1SK5R >1024 256 ND ND

1TR5R >1024 32 ND ND

Fractional inhibitory concentration index (FICI) as a measure of efficacy of combinatory therapy according to the following formula: FICI = (MIC of meropenem in combination/MIC of meropenem alone)+( MIC of colistin in combination/MIC of colistin alone). FICI was interpreted as follows: FICI ≤ 0.5, synergism; 0.5 < FICI < 1, additive; 1 ≤ FICI < 2, indifference; and FICI ≥ 2, antagonism, ICU; intersive care unit.

**Table S3.** Synergistic effects of antibiotics and colistin combination against *Klebsiella pneumoniae* isolates from hospitalized patients (Contd).

Clinical isolates MIC (μg/ml) FICI Outcome

Combination Colistin Imipenem Colistin Imipenem

1NT4Ng/1 256 256 64 64 0.500 Synergistic

64 32 0.375 Synergistic

1NT6Ng/1 256 256 64 64 0.500 Synergistic

1NT6Tu/1 256 128 ND ND

1NT6R 256 128 64 32 0.500 Synergistic

1NT7R 256 256 64 64 0.500 Synergistic

64 16 0.375 Synergistic

64 8 0.312 Synergistic

64 4 0.281 Synergistic

32 32 0.375 Synergistic

1NT8Th 256 128 ND ND

1NT6Ng (CCU)/1 512 128 ND ND

1NT6Th (CCU)/1 256 128 ND ND

1NT6R (CCU) 256 128 ND ND

1SK5R >1024 128 ND ND

1TR5R >1024 32 ND ND

Fractional inhibitory concentration index (FICI) as a measure of efficacy of combinatory therapy according to the following formula: FICI = (MIC of imipenem combination/MIC of imipenem alone)+( MIC of colistin in combination/MIC of colistin alone). FICI was interpreted as follows FICI ≤ 0.5, synergism; 0.5 < FICI < 1, additive; 1 ≤ FICI < 2, indifference; and FICI ≥ 2, antagonism.

**Table S3.** Synergistic effects of antibiotics and colistin combination against *Klebsiella pneumoniae* isolates from hospitalized patients (Contd).

Clinical isolates MIC (μg/ml) FICI Outcome

Combination Colistin Levofloxacin Colistin Levofloxacin

1NT4Ng/1 256 512 64 128 0.500 Synergistic

64 64 0.375 Synergistic

64 32 0.312 Synergistic

64 16 0.281 Synergistic

32 128 0.375 Synergistic

32 64 0.250 Synergistic

32 32 0.187 Synergistic

16 128 0.312 Synergistic

16 64 0.187 Synergistic

16 32 0.125 Synergistic

1NT6Ng/1 256 512 64 128 0.500 Synergistic

64 64 0.375 Synergistic

64 32 0.312 Synergistic

32 128 0.375 Synergistic

32 64 0.250 Synergistic

32 32 0.187 Synergistic

16 128 0.312 Synergistic

16 64 0.187 Synergistic

1NT6Tu/1 256 512 64 128 0.500 Synergistic

64 64 0.375 Synergistic

64 32 0.312 Synergistic

64 16 0.281 Synergistic

64 8 0.265 Synergistic

32 128 0.375 Synergistic

32 64 0.250 Synergistic

32 32 0.187 Synergistic

32 16 0.156 Synergistic

32 8 0.140 Synergistic

16 128 0.312 Synergistic

16 64 0.187 Synergistic

16 32 0.125 Synergistic

16 16 0.093 Synergistic

1NT6R 256 512 64 128 0.500 Synergistic

64 64 0.375 Synergistic

64 32 0.312 Synergistic

32 128 0.375 Synergistic

32 64 0.250 Synergistic

32 32 0.187 Synergistic

16 128 0.312 Synergistic

16 64 0.187 Synergistic

16 32 0.125 Synergistic

1NT7R 256 512 64 128 0.500 Synergistic

64 64 0.375 Synergistic

64 32 0.312 Synergistic

32 128 0.375 Synergistic

32 64 0.250 Synergistic

32 32 0.187 Synergistic

16 128 0.312 Synergistic

16 64 0.187 Synergistic

16 32 0.125 Synergistic

1NT8Th 256 512 64 128 0.500 Synergistic

64 64 0.375 Synergistic

64 32 0.312 Synergistic

32 128 0.375 Synergistic

32 64 0.250 Synergistic

32 32 0.187 Synergistic

16 128 0.312 Synergistic

16 64 0.187 Synergistic

16 32 0.125 Synergistic

1NT6Ng (CCU)/1 512 64 128 16 0.500 Synergistic

128 8 0.375 Synergistic

128 2 0.281 Synergistic

64 16 0.375 Synergistic

64 8 0.250 Synergistic

32 16 0.312 Synergistic

1NT6Th (CCU)/1 256 64 64 16 0.500 Synergistic

64 8 0.375 Synergistic

64 4 0.312 Synergistic

64 2 0.281 Synergistic

32 16 0.375 Synergistic

32 8 0.250 Synergistic

16 16 0.312 Synergistic

1NT6R (CCU) 256 512 64 128 0.500 Synergistic

64 64 0.375 Synergistic

64 32 0.312 Synergistic

32 128 0.375 Synergistic

32 64 0.250 Synergistic

32 32 0.187 Synergistic

16 128 0.312 Synergistic

16 64 0.187 Synergistic

1SK5R >1024 0.50 256 0.12 0.490 Synergistic

128 0.12 0.365 Synergistic

64 0.12 0.302 Synergistic

32 0.12 0.271 Synergistic

16 0.12 0.255 Synergistic

1TR5R >1024 0.25 ND ND

Fractional inhibitory concentration index (FICI) as a measure of efficacy of combinatory therapy according to the following formula: FICI = (MIC of levofloxacin combination/MIC of levofloxacin alone)+( MIC of colistin in combination/MIC of colistin alone). FICI was interpreted as follows: FICI ≤ 0.5, synergism; 0.5 < FICI < 1, additive; 1 ≤ FICI < 2, indifference; and FICI ≥ 2, antagonism.

**Table S3.** Synergistic effects of antibiotics and colistin combination against *Klebsiella pneumoniae* isolates from hospitalized patients (Contd).

Clinical isolates MIC (μg/ml) FICI Outcome

Combination Colistin Ceftolozane Colistin Ceftolozane

1NT4Ng/1 256 >1024 64 256 0.500 Synergistic

64 128 0.375 Synergistic

1NT6Ng/1 256 >1024 64 256 0.500 Synergistic

64 128 0.375 Synergistic

64 64 0.312 Synergistic

64 32 0.265 Synergistic

64 16 0.253 Synergistic

1NT6Tu/1 256 >1024 64 256 0.500 Synergistic

64 128 0.375 Synergistic

64 64 0.312 Synergistic

64 32 0.281 Synergistic

64 16 0.265 Synergistic

32 256 0.375 Synergistic

32 128 0.250 Synergistic

32 64 0.187 Synergistic

1NT6R 256 >1024 64 256 0.500 Synergistic

64 128 0.375 Synergistic

64 64 0.312 Synergistic

64 32 0.281 Synergistic

64 16 0.265 Synergistic

64 8 0.257 Synergistic

1NT7R 256 >1024 64 256 0.500 Synergistic

64 128 0.375 Synergistic

64 64 0.312 Synergistic

64 32 0.281 Synergistic

64 16 0.265 Synergistic

64 8 0.257 Synergistic

1NT8Th 256 >1024 ND ND

1NT6Ng (CCU)/1 512 >1024 128 256 0.500 Synergistic

128 128 0.375 Synergistic

128 64 0.312 Synergistic

128 32 0.281 Synergistic

128 16 0.265 Synergistic

1NT6Th (CCU)/1 256 >1024 64 256 0.500 Synergistic

64 128 0.375 Synergistic

64 64 0.312 Synergistic

64 32 0.281 Synergistic

1NT6R (CCU) 256 >1024 ND ND

1SK5R >1024 4 ND ND

1TR5R >1024 1 ND ND

Fractional inhibitory concentration index (FICI) as a measure of efficacy of combinatory therapy according to the following formula: FICI = (MIC of ceftolozane-tazobactam in combination/MIC of ceftolozane-tazobactam alone)+( MIC of colistin in combination/MIC of colistin alone). FICI was interpreted as follows FICI ≤ 0.5, synergism; 0.5 < FICI < 1, additive; 1 ≤ FICI < 2, indifference; and FICI ≥ 2, antagonism.

**Table S3.** Synergistic effects of antibiotics and colistin combination against *Klebsiella pneumoniae* isolates from hospitalized patients (Contd).

Clinical isolates MIC (μg/ml) FICI Outcome

Combination

Colistin Minocycline Colistin Minocycline

1NT4Ng/1 256 128 64 32 0.500 Synergistic

64 16 0.375 Synergistic

64 8 0.312 Synergistic

64 4 0.281 Synergistic

64 2 0.265 Synergistic

32 32 0.375 Synergistic

32 16 0.250 Synergistic

32 8 0.187 Synergistic

32 4 0.156 Synergistic

32 2 0.140 Synergistic

16 32 0.312 Synergistic

16 16 0.187 Synergistic

16 8 0.125 Synergistic

16 4 0.093 Synergistic

1NT6Ng/1 256 128 64 32 0.500 Synergistic

64 16 0.375 Synergistic

64 8 0.312 Synergistic

64 4 0.281 Synergistic

64 2 0.265 Synergistic

32 32 0.375 Synergistic

32 16 0.250 Synergistic

32 8 0.187 Synergistic

32 4 0.156 Synergistic

32 2 0.140 Synergistic

16 32 0.312 Synergistic

16 16 0.187 Synergistic

16 8 0.125 Synergistic

16 4 0.093 Synergistic

1NT6Tu/1 256 128 64 32 0.500 Synergistic

64 16 0.375 Synergistic

64 8 0.312 Synergistic

64 4 0.281 Synergistic

64 2 0.265 Synergistic

32 32 0.375 Synergistic

32 16 0.250 Synergistic

32 8 0.187 Synergistic

32 4 0.156 Synergistic

32 2 0.140 Synergistic

16 32 0.312 Synergistic

16 16 0.187 Synergistic

16 8 0.125 Synergistic

16 4 0.093 Synergistic

1NT6R 256 128 64 32 0.500 Synergistic

64 16 0.375 Synergistic

64 8 0.312 Synergistic

64 4 0.281 Synergistic

64 2 0.265 Synergistic

32 32 0.375 Synergistic

32 16 0.250 Synergistic

32 8 0.187 Synergistic

32 4 0.156 Synergistic

32 2 0.140 Synergistic

16 32 0.312 Synergistic

16 16 0.187 Synergistic

16 8 0.125 Synergistic

16 4 0.093 Synergistic

1NT7R 256 128 64 32 0.500 Synergistic

64 16 0.375 Synergistic

64 8 0.312 Synergistic

64 4 0.281 Synergistic

64 2 0.265 Synergistic

32 32 0.375 Synergistic

32 16 0.250 Synergistic

32 8 0.187 Synergistic

32 4 0.156 Synergistic

32 2 0.140 Synergistic

16 32 0.312 Synergistic

16 16 0.187 Synergistic

16 8 0.125 Synergistic

16 4 0.093 Synergistic

1NT8Th 256 128 64 32 0.500 Synergistic

64 16 0.375 Synergistic

64 8 0.312 Synergistic

64 4 0.281 Synergistic

64 2 0.265 Synergistic

32 32 0.375 Synergistic

32 16 0.250 Synergistic

32 8 0.187 Synergistic

32 4 0.156 Synergistic

32 2 0.140 Synergistic

16 32 0.312 Synergistic

16 16 0.187 Synergistic

16 8 0.125 Synergistic

16 4 0.093 Synergistic

1NT6Ng (CCU)/1 512 32 128 8 0.500 Synergistic

128 4 0.375 Synergistic

128 2 0.312 Synergistic

128 1 0.281 Synergistic

64 8 0.375 Synergistic

64 4 0.250 Synergistic

64 2 0.187 Synergistic

64 1 0.156 Synergistic

32 8 0.312 Synergistic

32 4 0.187 Synergistic

32 2 0.125 Synergistic

32 1 0.093 Synergistic

16 8 0.281 Synergistic

16 4 0.156 Synergistic

16 2 0.093 Synergistic

8 8 0.265 Synergistic

8 4 0.140 Synergistic

1NT6Th (CCU)/1 256 32 64 8 0.500 Synergistic

64 4 0.375 Synergistic

64 2 0.312 Synergistic

32 8 0.375 Synergistic

32 4 0.250 Synergistic

32 2 0.187 Synergistic

16 8 0.312 Synergistic

16 4 0.187 Synergistic

16 2 0.125 Synergistic

8 8 0.281 Synergistic

8 4 0.156 Synergistic

8 2 0.093 Synergistic

1NT6R (CCU) 256 128 64 32 0.500 Synergistic

64 16 0.375 Synergistic

64 8 0.312 Synergistic

64 4 0.281 Synergistic

64 2 0.265 Synergistic

32 32 0.375 Synergistic

32 16 0.250 Synergistic

32 8 0.187 Synergistic

32 4 0.156 Synergistic

32 2 0.140 Synergistic

32 1 0.132 Synergistic

16 32 0.312 Synergistic

16 16 0.187 Synergistic

16 8 0.125 Synergistic

16 4 0.093 Synergistic

16 2 0.078 Synergistic

8 32 0.281 Synergistic

8 16 0.156 Synergistic

8 8 0.093 Synergistic

8 4 0.062 Synergistic

1SK5R >1024 16 ND ND

1TR5R >1024 0.25 ND ND

Fractional inhibitory concentration index (FICI) as a measure of efficacy of combinatory therapy according to the following formula: FICI = (MIC of minocycline combination/MIC of minocycline alone)+( MIC of colistin in combination/MIC of colistin alone). FICI was interpreted as follows: FICI ≤ 0.5, synergism; 0.5 < FICI < 1, additive; 1 ≤ FICI < 2, indifference; and FICI ≥ 2, antagonism.

**Table S3.** Synergistic effects of antibiotics and colistin combination against *Klebsiella pneumoniae* isolates from hospitalized patients (Contd).

Clinical isolates MIC (μg/ml) FICI Outcome

Combination Colistin Tobramycin Colistin Tobramycin

1NT4Ng/1 256 32 64 8 0.500 Synergistic

1NT6Ng/1 256 32 64 8 0.500 Synergistic

1NT6Tu/1 256 64 ND ND

1NT6R 256 64 64 16 0.500 Synergistic

32 16 0.375 Synergistic

1NT7R 256 32 ND ND

1NT8Th 256 32 ND ND

1NT6Ng (CCU)/1 512 16 128 4 0.500 Synergistic

128 2 0.375 Synergistic

64 4 0.375 Synergistic

1NT6Th (CCU)/1 256 16 64 4 0.500 Synergistic

64 2 0.375 Synergistic

64 1 0.312 Synergistic

32 4 0.375 Synergistic

16 4 0.312 Synergistic

1NT6R (CCU) 256 32 ND ND

1SK5R >1024 8 ND ND

1TR5R >1024 1 ND ND

Fractional inhibitory concentration index (FICI) as a measure of efficacy of combinatory therapy according to the following formula: FICI = (MIC of tobramycin combination/MIC of tobramycin alone)+( MIC of colistin in combination/MIC of colistin alone). FICI was interpreted as follows: FICI ≤ 0.5, synergism; 0.5 < FICI < 1, additive; 1 ≤ FICI < 2, indifference; and FICI ≥ 2, antagonism.

**Table S3.** Synergistic effects of antibiotics and colistin combination against *Klebsiella pneumoniae* isolates from hospitalized patients (Contd).

Clinical isolates MIC (μg/ml) FICI Outcome

Combination Colistin Cefotaxime Colistin Cefotaxime

1NT4Ng/1 256 >1024 64 256 0.500 Synergistic

64 128 0.375 Synergistic

32 256 0.375 Synergistic

16 256 0.312 Synergistic

1NT6Ng/1 256 >1024 64 256 0.500 Synergistic

32 256 0.375 Synergistic

16 256 0.312 Synergistic

1NT6Tu/1 256 >1024 64 256 0.500 Synergistic

64 128 0.375 Synergistic

64 64 0.312 Synergistic

32 256 0.375 Synergistic

16 256 0.312 Synergistic

1NT6R 256 >1024 64 256 0.500 Synergistic

64 128 0.375 Synergistic

32 256 0.375 Synergistic

16 256 0.312 Synergistic

1NT7R 256 >1024 64 256 0.500 Synergistic

64 128 0.375 Synergistic

64 64 0.312 Synergistic

32 256 0.375 Synergistic 32 128 0.250 Synergistic

32 64 0.187 Synergistic

16 256 0.312 Synergistic

1NT8Th 256 >1024 64 256 0.500 Synergistic

64 128 0.375 Synergistic

64 64 0.312 Synergistic

32 256 0.375 Synergistic

16 256 0.312 Synergistic

1NT6Ng (CCU)/1 512 >1024 128 256 0.500 Synergistic

64 256 0.375 Synergistic

32 256 0.312 Synergistic

16 256 0.281 Synergistic

1NT6Th (CCU)/1 256 >1024 64 256 0.500 Synergistic

64 128 0.375 Synergistic

32 256 0.375 Synergistic

16 256 0.312 Synergistic

1NT6R (CCU) 256 >1024 64 256 0.500 Synergistic

64 128 0.375 Synergistic

32 256 0.375 Synergistic

32 128 0.250 Synergistic 16 256 0.312 Synergistic

1SK5R >1024 512 ND ND

1TR5R >1024 4 ND ND

Fractional inhibitory concentration index (FICI) as a measure of efficacy of combinatory therapy according to the following formula: FICI = (MIC of cefotaxime combination/MIC of cefotaxime alone)+( MIC of colistin in combination/MIC of colistin alone). FICI was interpreted as follows: FICI ≤ 0.5, synergism; 0.5 < FICI < 1, additive; 1 ≤ FICI < 2, indifference; and FICI ≥ 2, antagonism.

**Table S3.** Synergistic effects of antibiotics and colistin combination against *Klebsiella pneumoniae* isolates from hospitalized patients (Contd).

Clinical isolates MIC (μg/ml) FICI Outcome

Combination Colistin Cefoperazone Colistin Cefoperazone

1NT4Ng/1 256 >1024 64 256 0.500 Synergistic

32 256 0.375 Synergistic

1NT6Ng/1 256 >1024 64 256 0.500 Synergistic

32 256 0.375 Synergistic

1NT6Tu/1 256 >1024 64 256 0.500 Synergistic

64 128 0.375 Synergistic

32 256 0.375 Synergistic

16 256 0.312 Synergistic

1NT6R 256 >1024 64 256 0.500 Synergistic

32 256 0.375 Synergistic

16 256 0.312 Synergistic

1NT7R 256 >1024 64 256 0.500 Synergistic

64 128 0.375 Synergistic

64 64 0.312 Synergistic

32 256 0.375 Synergistic

16 256 0.312 Synergistic

1NT8Th 256 >1024 64 256 0.500 Synergistic

64 128 0.375 Synergistic

64 64 0.312 Synergistic

64 32 0.281 Synergistic

32 256 0.375 Synergistic

16 256 0.312 Synergistic

1NT6Ng (CCU)/1 512 >1024 128 256 0.500 Synergistic

64 256 0.375 Synergistic

1NT6Th (CCU)/1 256 >1024 64 256 0.500 Synergistic

64 128 0.375 Synergistic

64 64 0.312 Synergistic

32 256 0.375 Synergistic

16 256 0.312 Synergistic

1NT6R (CCU) 256 >1024 64 256 0.500 Synergistic

32 256 0.375 Synergistic

16 256 0.312 Synergistic

1SK5R >1024 4 ND ND

1TR5R >1024 2 ND ND

Fractional inhibitory concentration index (FICI) as a measure of efficacy of combinatory therapy according to the following formula: FICI = (MIC of cefoperazone combination/MIC of cefoperazone alone)+( MIC of colistin in combination/MIC of colistin alone). FICI was interpreted as follows: FICI ≤ 0.5, synergism; 0.5 < FICI < 1, additive; 1 ≤ FICI < 2, indifference; and FICI ≥ 2, antagonism.

**Table S3.** Synergistic effects of antibiotics and colistin combination against *Klebsiella pneumoniae* isolates from hospitalized patients (Contd).

Clinical isolates MIC (μg/ml) FICI Outcome

Combination

Colistin Moxifloxacin Colistin Moxifloxacin

1NT4Ng/1 256 512 64 128 0.500 Synergistic

64 64 0.375 Synergistic

64 32 0.312 Synergistic

64 16 0.281 Synergistic

32 128 0.375 Synergistic

32 64 0.250 Synergistic

32 32 0.187 Synergistic

32 16 0.156 Synergistic

16 128 0.312 Synergistic

16 64 0.187 Synergistic

16 32 0.125 Synergistic

8 128 0.281 Synergistic

8 64 0.156 Synergistic

8 32 0.093 Synergistic

1NT6Ng/1 256 512 64 128 0.500 Synergistic

64 64 0.375 Synergistic

64 32 0.312 Synergistic

64 16 0.281 Synergistic

32 128 0.375 Synergistic

32 64 0.250 Synergistic

32 32 0.187 Synergistic

32 16 0.156 Synergistic

16 128 0.312 Synergistic

16 64 0.187 Synergistic

16 32 0.125 Synergistic

8 128 0.281 Synergistic

8 64 0.156 Synergistic

8 32 0.093 Synergistic

1NT6Tu/1 256 512 64 128 0.500 Synergistic

64 64 0.375 Synergistic

64 32 0.312 Synergistic

64 16 0.281 Synergistic

32 128 0.375 Synergistic

32 64 0.250 Synergistic

32 32 0.187 Synergistic

32 16 0.156 Synergistic

16 128 0.312 Synergistic

16 64 0.187 Synergistic

16 32 0.125 Synergistic

8 128 0.281 Synergistic

8 64 0.156 Synergistic

8 32 0.093 Synergistic

1NT6R 256 512 64 128 0.500 Synergistic

64 64 0.375 Synergistic

64 32 0.312 Synergistic

64 16 0.281 Synergistic

32 128 0.375 Synergistic

32 64 0.250 Synergistic

32 32 0.187 Synergistic

32 16 0.156 Synergistic

16 128 0.312 Synergistic

16 64 0.187 Synergistic

16 32 0.125 Synergistic

8 128 0.281 Synergistic

8 64 0.156 Synergistic

8 32 0.093 Synergistic

1NT7R 256 512 64 128 0.500 Synergistic

64 64 0.375 Synergistic

64 32 0.312 Synergistic

64 16 0.281 Synergistic

32 128 0.375 Synergistic

32 64 0.250 Synergistic

32 32 0.187 Synergistic

32 16 0.156 Synergistic

16 128 0.312 Synergistic

16 64 0.187 Synergistic

16 32 0.125 Synergistic

1NT8Th 256 512 64 128 0.500 Synergistic

64 64 0.375 Synergistic

64 32 0.312 Synergistic

64 16 0.281 Synergistic

64 8 0.265 Synergistic

32 128 0.375 Synergistic

32 64 0.250 Synergistic

32 32 0.187 Synergistic

32 16 0.156 Synergistic

16 128 0.312 Synergistic

16 64 0.187 Synergistic

16 32 0.125 Synergistic

16 16 0.093 Synergistic

8 128 0.281 Synergistic

8 64 0.156 Synergistic

1NT6Ng (CCU)/1 512 32 128 8 0.500 Synergistic

128 4 0.375 Synergistic

128 2 0.312 Synergistic

64 8 0.375 Synergistic

1NT6Th (CCU)/1 256 32 64 8 0.500 Synergistic

64 4 0.375 Synergistic

64 2 0.312 Synergistic

64 1 0.281 Synergistic

32 8 0.375 Synergistic

32 4 0.250 Synergistic

32 2 0.187 Synergistic

32 1 0.156 Synergistic

16 8 0.312 Synergistic

8 8 0.281 Synergistic

1NT6R (CCU) 256 512 64 128 0.500 Synergistic

64 64 0.375 Synergistic

64 32 0.312 Synergistic

64 16 0.281 Synergistic

32 128 0.375 Synergistic

32 64 0.250 Synergistic

32 32 0.187 Synergistic

32 16 0.156 Synergistic

16 128 0.312 Synergistic

16 64 0.187 Synergistic

16 32 0.125 Synergistic

16 16 0.093 Synergistic

8 128 0.281 Synergistic

8 64 0.156 Synergistic

8 32 0.093 Synergistic

1SK5R >1024 4 ND ND

1TR5R >1024 4 ND ND

Fractional inhibitory concentration index (FICI) as a measure of efficacy of combinatory therapy according to the following formula: FICI = (MIC of moxifloxacin combination/MIC of moxifloxacin alone)+( MIC of colistin in combination/MIC of colistin alone). FICI was interpreted as follows: FICI ≤ 0.5, synergism; 0.5 < FICI < 1, additive; 1 ≤ FICI < 2, indifference; and FICI ≥ 2, antagonism.

**Table S3.** Synergistic effects of antibiotics and colistin combination against *Klebsiella pneumoniae* isolates from hospitalized patients (Contd).

Clinical isolates MIC (μg/ml) FICI Outcome

Combination Colistin Amikacin Colistin Amikacin

1NT4Ng/1 256 16 64 4 0.500 Synergistic

32 4 0.375 Synergistic

16 4 0.312 Synergistic

8 4 0.281 Synergistic

1NT6Ng/1 256 16 64 4 0.500 Synergistic

64 2 0.375 Synergistic

64 1 0.312 Synergistic

32 4 0.375 Synergistic

32 2 0.250 Synergistic

16 4 0.312 Synergistic

8 4 0.281 Synergistic

1NT6Tu/1 256 8 64 2 0.500 Synergistic

64 1 0.375 Synergistic

64 0.50 0.312 Synergistic

64 0.25 0.281 Synergistic

64 0.12 0.265 Synergistic

1NT6R 256 16 64 4 0.500 Synergistic

64 2 0.375 Synergistic

64 1 0.312 Synergistic

64 0.50 0.281 Synergistic

64 0.25 0.265 Synergistic

32 4 0.375 Synergistic

32 2 0.250 Synergistic

32 1 0.180 Synergistic

32 0.50 0.156 Synergistic

32 0.25 0.140 Synergistic

16 4 0.312 Synergistic

16 2 0.187 Synergistic

16 1 0.125 Synergistic

16 0.50 0.093 Synergistic

16 0.25 0.078 Synergistic

8 4 0.281 Synergistic

1NT7R 256 8 ND ND

1NT8Th 256 16 64 4 0.500 Synergistic

32 4 0.375 Synergistic

16 4 0.312 Synergistic

1NT6Ng (CCU)/1 512 16 128 4 0.500 Synergistic

128 2 0.375 Synergistic

128 1 0.312 Synergistic

64 4 0.375 Synergistic

32 4 0.312 Synergistic

16 4 0.281 Synergistic

1NT6Th (CCU)/1 256 16 64 4 0.500 Synergistic

64 2 0.375 Synergistic

64 1 0.312 Synergistic

64 0.50 0.281 Synergistic

64 0.25 0.265 Synergistic

64 0.12 0.257 Synergistic

64 0.06 0.253 Synergistic

32 4 0.375 Synergistic

32 2 0.250 Synergistic

32 1 0.187 Synergistic

32 0.50 0.156 Synergistic

32 0.25 0.140 Synergistic

32 0.12 0.132 Synergistic

16 4 0.312 Synergistic

16 2 0.187 Synergistic

16 1 0.125 Synergistic

16 0.50 0.093 Synergistic

16 0.25 0.078 Synergistic

16 0.12 0.070 Synergistic

8 4 0.281 Synergistic

8 2 0.156 Synergistic

8 1 0.093 Synergistic

8 0.50 0.062 Synergistic

1NT6R (CCU) 256 16 64 4 0.500 Synergistic

64 2 0.375 Synergistic

32 4 0.375 Synergistic

32 2 0.250 Synergistic

16 4 0.312 Synergistic

8 4 0.281 Synergistic

1SK5R >1024 4 ND ND

1TR5R >1024 4 ND ND

Fractional inhibitory concentration index (FICI) as a measure of efficacy of combinatory therapy according to the following formula: FICI = (MIC of amikacin combination/MIC of amikacin alone)+( MIC of colistin in combination/MIC of colistin alone). FICI was interpreted as follows: FICI ≤ 0.5, synergism; 0.5 < FICI < 1, additive; 1 ≤ FICI < 2, indifference; and FICI ≥ 2, antagonism.

**Table S3.** Synergistic effects of antibiotics and colistin combination against *Klebsiella pneumoniae* isolates from hospitalized patients (Contd).

Clinical isolates MIC (μg/ml) FICI Outcome

Combination Colistin Gentamicin Colistin Gentamicin

1NT4Ng/1 256 128 64 32 0.500 Synergistic

64 16 0.375 Synergistic

32 32 0.375 Synergistic

1NT6Ng/1 256 128 64 32 0.500 Synergistic

64 16 0.375 Synergistic

64 8 0.312 Synergistic

64 4 0.281 Synergistic

32 32 0.375 Synergistic

32 16 0.250 Synergistic

16 32 0.312 Synergistic

16 16 0.187 Synergistic

8 32 0.281 Synergistic

1NT6Tu/1 256 256 64 64 0.500 Synergistic

64 32 0.375 Synergistic

64 16 0.312 Synergistic

64 8 0.281 Synergistic

64 4 0.265 Synergistic

32 64 0.375 Synergistic

16 64 0.312 Synergistic

8 64 0.281 Synergistic

1NT6R 256 128 64 32 0.500 Synergistic

64 16 0.375 Synergistic

64 8 0.312 Synergistic

64 4 0.281 Synergistic

32 32 0.375 Synergistic

32 16 0.250 Synergistic

32 8 0.187 Synergistic

32 4 0.156 Synergistic

16 32 0.312 Synergistic

16 16 0.187 Synergistic

16 8 0.125 Synergistic

1NT7R 256 128 64 32 0.500 Synergistic

64 16 0.375 Synergistic

32 32 0.375 Synergistic

1NT8Th 256 64 64 16 0.500 Synergistic

1NT6Ng (CCU)/1 512 128 128 32 0.500 Synergistic

128 16 0.375 Synergistic

128 8 0.312 Synergistic

64 32 0.375 Synergistic

64 16 0.250 Synergistic

32 8 0.312 Synergistic

1NT6Th (CCU)/1 256 128 64 32 0.500 Synergistic

64 16 0.375 Synergistic

32 32 0.375 Synergistic

1NT6R (CCU) 256 128 64 32 0.500 Synergistic

64 16 0.375 Synergistic

64 8 0.312 Synergistic

64 4 0.281 Synergistic

32 32 0.375 Synergistic

32 16 0.250 Synergistic

32 8 0.187 Synergistic

16 32 0.312 Synergistic

1SK5R >1024 4 ND ND

1TR5R >1024 0.5 ND ND

Fractional inhibitory concentration index (FICI) as a measure of efficacy of combinatory therapy according to the following formula: FICI = (MIC of gentamicin combination/MIC of gentamicin alone)+( MIC of colistin in combination/MIC of colistin alone). FICI was interpreted as follows: FICI ≤ 0.5, synergism; 0.5 < FICI < 1, additive; 1 ≤ FICI < 2, indifference; and FICI ≥ 2, antagonism.

**Table S3.** Synergistic effects of antibiotics and colistin combination against *Klebsiella pneumoniae* isolates from hospitalized patients (Contd).

Clinical isolates MIC (μg/ml) FICI Outcome

Combination Colistin Ceftazidime Colistin Ceftazidime

1NT4Ng/1 256 >1024 64 256 0.500 Synergistic

64 128 0.375 Synergistic

32 256 0.375 Synergistic

16 256 0.312 Synergistic

1NT6Ng/1 256 >1024 64 256 0.500 Synergistic

64 128 0.375 Synergistic

32 256 0.375 Synergistic

1NT6Tu/1 256 >1024 64 256 0.500 Synergistic

32 256 0.375 Synergistic

16 256 0.312 Synergistic

1NT6R 256 >1024 64 256 0.500 Synergistic

64 128 0.375 Synergistic

32 256 0.375 Synergistic

16 256 0.312 Synergistic

1NT7R 256 >1024 64 256 0.500 Synergistic

64 128 0.375 Synergistic

32 256 0.375 Synergistic

32 128 0.250 Synergistic

16 256 0.312 Synergistic

1NT8Th 256 >1024 64 256 0.500 Synergistic

64 128 0.375 Synergistic

64 64 0.312 Synergistic

32 256 0.375 Synergistic

32 128 0.250 Synergistic

16 256 0.312 Synergistic

16 128 0.187 Synergistic

1NT6Ng (CCU)/1 512 >1024 128 256 0.500 Synergistic

128 128 0.375 Synergistic

128 64 0.312 Synergistic

128 32 0.281 Synergistic

128 16 0.265 Synergistic

64 256 0.375 Synergistic

64 128 0.250 Synergistic

64 64 0.187 Synergistic

32 256 0.312 Synergistic

32 128 0.187 Synergistic

16 256 0.281 Synergistic

1NT6Th (CCU)/1 256 >1024 64 256 0.500 Synergistic

64 128 0.375 Synergistic

64 64 0.312 Synergistic

64 32 0.281 Synergistic

32 256 0.375 Synergistic

32 128 0.250 Synergistic

16 256 0.312 Synergistic

1NT6R (CCU) 256 >1024 64 256 0.500 Synergistic

64 128 0.375 Synergistic

32 256 0.375 Synergistic

16 256 0.312 Synergistic

1SK5R >1024 256 ND ND

1TR5R >1024 0.25 ND ND

Fractional inhibitory concentration index (FICI) as a measure of efficacy of combinatory therapy according to the following formula: FICI = (MIC of ceftazidime combination/MIC of ceftazidime alone)+( MIC of colistin in combination/MIC of colistin alone). FICI was interpreted as follows: FICI ≤ 0.5, synergism; 0.5 < FICI < 1, additive; 1 ≤ FICI < 2, indifference; and FICI ≥ 2, antagonism.

**Table S3.** Synergistic effects of antibiotics and colistin combination against *Klebsiella pneumoniae* isolates from hospitalized patients (Contd).

Clinical isolates MIC (μg/ml) FICI Outcome

Combination Colistin Tigecycline Colistin Tigecycline

1NT4Ng/1 256 8 ND ND

1NT6Ng/1 256 8 ND ND

1NT6Tu/1 256 8 ND ND

1NT6R 256 8 ND ND

1NT7R 256 8 ND ND

1NT8Th 256 8 ND ND

1NT6Ng (CCU)/1 512 8 ND ND

1NT6Th (CCU)/1 256 8 ND ND

1NT6R (CCU) 256 8 ND ND

1SK5R >1024 16 ND ND

1TR5R >1024 8 ND ND

Fractional inhibitory concentration index (FICI) as a measure of efficacy of combinatory therapy according to the following formula: FICI = (MIC of tigecycline combination/MIC of tigecycline alone)+( MIC of colistin in combination/MIC of colistin alone). FICI was interpreted as follows: FICI ≤ 0.5, synergism; 0.5 < FICI < 1, additive; 1 ≤ FICI < 2, indifference; and FICI ≥ 2, antagonism..

**Table S3.** Synergistic effects of antibiotics and colistin combination against *Klebsiella pneumoniae* isolates from hospitalized patients (Contd).

Clinical isolates MIC (μg/ml) FICI Outcome

Combination Colistin Ciprofloxacin Colistin Ciprofloxacin

1NT4Ng/1 256 >256 64 64 0.500 Synergistic

64 32 0.375 Synergistic

64 16 0.312 Synergistic

64 8 0.281 Synergistic

64 4 0.265 Synergistic

64 2 0.257 Synergistic

1NT6Ng/1 256 >256 64 64 0.500 Synergistic

64 32 0.375 Synergistic

64 16 0.312 Synergistic

64 8 0.281 Synergistic

64 4 0.265 Synergistic

64 2 0.257 Synergistic

1NT6Tu/1 256 >256 ND ND

1NT6R 256 >256 64 64 0.500 Synergistic

32 64 0.375 Synergistic

1NT7R 256 >256 64 64 0.500 Synergistic

1NT8Th 256 >256 64 64 0.500 Synergistic

1NT6Ng (CCU)/1 512 128 ND ND

1NT6Th (CCU)/1 256 128 ND ND

1NT6R (CCU) 256 256 ND ND

1SK5R >1024 32 ND ND

1TR5R >1024 4 ND ND

Fractional inhibitory concentration index (FICI) as a measure of efficacy of combinatory therapy according to the following formula: FICI = (MIC of ciprofloxacin combination/MIC of ciprofloxacin alone)+( MIC of colistin in combination/MIC of colistin alone). FICI was interpreted as follows: FICI ≤ 0.5, synergism; 0.5 < FICI < 1, additive; 1 ≤ FICI < 2, indifference; and FICI ≥ 2, antagonism.

**Table S3.** Synergistic effects of antibiotics and colistin combination against *Klebsiella pneumoniae* isolates from hospitalized patients (Contd).

Clinical isolates MIC (μg/ml) FICI Outcome

Combination Colistin Fosfomycin Colistin Fosfomycin

1NT4Ng/1 256 >1024 64 256 0.500 Synergistic

1NT6Ng/1 256 >1024 64 256 0.500 Synergistic

64 128 0.375 Synergistic

1NT6Tu/1 256 >1024 ND ND

1NT6R 256 >1024 ND ND

1NT7R 256 >1024 64 256 0.500 Synergistic

1NT8Th 256 >1024 64 256 0.500 Synergistic

1NT6Ng (CCU)/1 512 128 128 32 0.500 Synergistic

128 16 0.375 Synergistic

128 8 0.312 Synergistic

128 4 0.281 Synergistic

128 2 0.265 Synergistic

128 1 0.257 Synergistic

64 32 0.375 Synergistic

64 16 0.250 Synergistic

64 8 0.187 Synergistic

64 4 0.156 Synergistic

64 2 0.140 Synergistic

32 32 0.312 Synergistic

32 16 0.187 Synergistic

32 8 0.125 Synergistic

32 4 0.093 Synergistic

32 2 0.078 Synergistic

16 32 0.281 Synergistic

16 16 0.156 Synergistic

1NT6Th (CCU)/1 256 128 64 32 0.500 Synergistic

64 16 0.375 Synergistic

64 8 0.312 Synergistic

64 4 0.281 Synergistic

64 2 0.265 Synergistic

32 32 0.375 Synergistic

32 16 0.250 Synergistic

32 8 0.187 Synergistic

32 4 0.156 Synergistic

32 2 0.140 Synergistic

16 16 0.187 Synergistic

16 8 0.125 Synergistic

16 4 0.093 Synergistic

8 16 0.156 Synergistic

8 8 0.093 Synergistic

8 4 0.062 Synergistic

1NT6R (CCU) 256 >1024 64 256 0.500 Synergistic

32 256 0.375 Synergistic

1SK5R >1024 32 ND ND

1TR5R >1024 0.5 256 0.125 0.500 Synergistic

Fractional inhibitory concentration index (FICI) as a measure of efficacy of combinatory therapy according to the following formula: FICI = (MIC of fosfomycin combination/MIC of fosfomycin alone)+( MIC of colistin in combination/MIC of colistin alone). FICI was interpreted as follows: FICI ≤ 0.5, synergism; 0.5 < FICI < 1, additive; 1 ≤ FICI < 2, indifference; and FICI ≥ 2, antagonism.

**Table S3.** Synergistic effects of antibiotics and colistin combination against *Klebsiella pneumoniae* isolates from hospitalized patients (Contd).

Clinical isolates MIC (μg/ml) FICI Outcome

Combination Colistin Piperacillin Colistin Piperacillin

1NT4Ng/1 256 >1024 64 256 0.500 Synergistic

64 128 0.375 Synergistic

64 64 0.312 Synergistic

64 32 0.281 Synergistic

64 16 0.265 Synergistic

64 8 0.257 Synergistic

64 4 0.253 Synergistic

64 2 0.251 Synergistic

64 1 0.250 Synergistic

32 256 0.375 Synergistic

32 128 0.250 Synergistic

32 64 0.187 Synergistic

32 32 0.156 Synergistic

32 16 0.140 Synergistic

32 8 0.132 Synergistic

32 4 0.128 Synergistic

1NT6Ng/1 256 >1024 64 256 0.500 Synergistic

64 128 0.375 Synergistic

64 64 0.312 Synergistic

64 32 0.281 Synergistic

64 16 0.265 Synergistic

64 8 0.257 Synergistic

64 4 0.253 Synergistic

64 2 0.251 Synergistic

64 1 0.250 Synergistic

32 256 0.375 Synergistic

32 128 0.250 Synergistic

32 64 0.187 Synergistic

1NT6Tu/1 256 >1024 64 256 0.500 Synergistic

64 128 0.375 Synergistic

64 64 0.312 Synergistic

64 32 0.281 Synergistic

64 16 0.265 Synergistic

64 8 0.257 Synergistic

64 4 0.253 Synergistic

64 2 0.251 Synergistic

64 1 0.250 Synergistic

32 256 0.375 Synergistic

32 128 0.250 Synergistic

32 64 0.187 Synergistic

32 32 0.156 Synergistic

32 16 0.140 Synergistic

32 8 0.132 Synergistic

32 4 0.128 Synergistic

32 2 0.126 Synergistic

1NT6R 256 >1024 64 256 0.500 Synergistic

64 128 0.375 Synergistic

64 64 0.312 Synergistic

64 32 0.281 Synergistic

64 16 0.265 Synergistic

64 8 0.257 Synergistic

64 4 0.253 Synergistic

64 2 0.251 Synergistic

1NT7R 256 >1024 ND ND

1NT8Th 256 >1024 64 256 0.500 Synergistic

64 128 0.375 Synergistic

64 64 0.312 Synergistic

64 32 0.281 Synergistic

64 16 0.265 Synergistic

64 8 0.257 Synergistic

64 4 0.253 Synergistic

64 2 0.251 Synergistic

1NT6Ng (CCU)/1 512 >1024 128 256 0.500 Synergistic

128 128 0.375 Synergistic

128 64 0.312 Synergistic

128 32 0.281 Synergistic

128 16 0.265 Synergistic

64 256 0.375 Synergistic

64 128 0.250 Synergistic

64 64 0.187 Synergistic

64 32 0.251 Synergistic

64 16 0.156 Synergistic

32 256 0.312 Synergistic

32 128 0.250 Synergistic

32 64 0.187 Synergistic

32 32 0.093 Synergistic

32 16 0.078 Synergistic

16 256 0.281 Synergistic

16 128 0.156 Synergistic

16 64 0.093 Synergistic

16 32 0.062 Synergistic

16 16 0.046 Synergistic

1NT6Th (CCU)/1 256 >1024 64 256 0.500 Synergistic

64 128 0.375 Synergistic

64 64 0.312 Synergistic

64 32 0.281 Synergistic

64 16 0.265 Synergistic

64 8 0.257 Synergistic

64 4 0.253 Synergistic

64 2 0.251 Synergistic

64 1 0.250 Synergistic

32 256 0.375 Synergistic

32 128 0.250 Synergistic

32 64 0.187 Synergistic

32 32 0.156 Synergistic

32 16 0.140 Synergistic

32 8 0.132 Synergistic

32 4 0.128 Synergistic

32 2 0.126 Synergistic

16 256 0.312 Synergistic

16 128 0.187 Synergistic

16 64 0.125 Synergistic

16 32 0.093 Synergistic

16 16 0.078 Synergistic

16 8 0.070 Synergistic

16 4 0.066 Synergistic

1NT6R (CCU) 256 >1024 64 256 0.500 Synergistic

64 128 0.375 Synergistic

64 64 0.312 Synergistic

64 32 0.281 Synergistic

64 16 0.265 Synergistic

64 8 0.257 Synergistic

64 4 0.253 Synergistic

64 2 0.251 Synergistic

1SK5R >1024 16 ND ND

1TR5R >1024 0.5 256 0.125 0.500 Synergistic

128 0.125 0.375 Synergistic

Fractional inhibitory concentration index (FICI) as a measure of efficacy of combinatory therapy according to the following formula: FICI = (MIC of piperacillin combination/MIC of piperacillin alone)+( MIC of colistin in combination/MIC of colistin alone). FICI was interpreted as follows: FICI ≤ 0.5, synergism; 0.5 < FICI < 1, additive; 1 ≤ FICI < 2, indifference; and FICI ≥ 2, antagonism.
