# Supplementary material for: Synergistic antibacterial effects of colistin in combination with aminoglycoside, carbapenems, cephalosporins, fluoroquinolones, tetracyclines, fosfomycin, and piperacillin on multidrug resistant Klebsiella pneumoniae isolates
Source: PLoS One. 2021 Jan 6;16(1):e0244673. doi: 10.1371/journal.pone.0244673 (PMC7787437; doi:10.1371/journal.pone.0244673)
Supplement: Attachment — Submitted filename: Response to reviewers comment.docx [file pone.0244673.s004.docx]

5. Review Comments to the Author

Please use the space provided to explain your answers to the questions above. You may also include additional comments for the author, including concerns about dual publication, research ethics, or publication ethics. (Please upload your review as an attachment if it exceeds 20,000 characters)

**Reviewer #1**:

The scientific work described in the manuscript is original, the research has been conducted using standard experimental techniques and references. The data has been analyzed and presented. The findings of the research can be applicable in the patient care. Some of the revisions suggested-
Introduction
Line 4 Infectious- infections

**Corrected**
Line 6 Enterobacteriaceae- Enterobacteriaceae member

**Corrected**
Line 10 bacteria isolates- bacterial isolates

**Corrected**
Line 12- Gram-negative bacteria, colistin competitively displaces

**Corrected**
Line 14- Colistin resistance in Enterobacteriaceae

**Corrected**
What is the meaning of collection of antibiotics? Clarify

**Clarification added.**

The resistant isolates were further tested against several groups of antibiotics, and the synergistic antibacterial effects of colistin combinations with other antibiotics were evaluated.

Mention the limitation of this research work

The study presents *In vitro* experimental antibacterial data from broth micro-dilution technique and the checkerboard assay, but did not monitor the time-kill.

**Reviewer #2:**

Dear Authors
The paper describes the synergistic effect of colistin with antibiotics of different classes such as cephalosporins, carbapenems, fluoroquinalones, tetracyclins, fosfomycin, piperacillin tazobactam, among others.
I request the authors to clarify on the following aspects
1. The breakpoints for colistin changed in 2020 both by CLSI and EUCAST to Intermediate and Resistant. Have the authors considering this change and has the data been interpreted in light of this change. If not, Why have the authors presented data with the old breakpoints

**YES, the new CLSI and EUCAST documents were considered in the interpretations.**

The CLSI 2020 document presented a colistin break point of ≤ 2 as intermediate and ≥ 4 as resistant, whereas the EUCAST 2020 documents presents colistin breakpoints of ≤ 2 as susceptible and > 2 as resistant. Thus, we believe that colistin breakpoint as defined in the manuscript (≤ 2 as susceptible) was in line with the recommended interpretations.

2. All the isolates studied from different centers seem to be colonizers like in the nasophyrynx, throat, rectal, ET tube and environmental cultures. Why were these organisms cultured and reported from these sites.

The study protocol included surveillance data of colonization of carbapenem-resistant Enterobacteriaceae in the patients who admitted in the intensive care units of each hospital. We obtained the specimens from the potential sites of colonization including throat, rectum, endotracheal tube (among patients with mechanical ventilator), gastric content (among the patients with oro/nasogastric tube) and hospital environment of the patients (bed sheet, pillowcase and bed rail).

What was the significance of these isolates clinically? Was an outbreak being studied?

Or were these organisms in a special subset of patients. What is the clinical relevance of these isolates?

According to the rising prevalence of carbapenem-resistant Enterobacteriaceae in intensive care units (ICUs) of hospitals in Southern Thailand, a prospective epidemiological study was conducted to determine characteristics, risk factors and clinical outcomes of patients admitted in a network of hospitals including Songklanagarind Hospital, a university hospital with referral centres at 3 tertiary care hospital (Yala, Trang, and Hat Yai Hospital) and 5 provincial hospitals (Pattani, Narathiwat, Phatthalung, Satun, and Songkhla Hospital). We randomly selected only one isolates of *K. pneumoniae* from patients with multiple sites of colonization and only one isolate for the patients with multiple sites of environmental contamination.

3. How was the MIC for fosfomycin done. The recommended method is agar dilution. Why was broth dilution used instead?

Although the agar dilution method is recommended as reference method for the determination of Fosfomycin MICs, using the reference agar dilution method in a checkerboard analysis is practically difficult. Thus, the broth microdilution with glucose-6-phosphate (G-6-P) was used [28].

28.	Flamm RK, Rhomberg PR, Lindley JM, Sweeney K, Ellis-Grosse E, Shortridge D. Evaluation of the bactericidal activity of fosfomycin in combination with selected antimicrobial comparison agents tested against gram-negative bacterial strains by using time-kill curves. Antimicrobial Agents and Chemotherapy. 2019;63(5).

4. MBC results need to be explained better as Time Kill studies are the gold standard rather than the checkerboard method for synergy testing

**MBC methodology added.**

To determine the MBC, MIC and supra-MIC dilutions were spotted on an agar plate and incubated overnight at 37 °C. Bacterial growth was observed, and MBC was defined as the lowest concentration that showed no visible bacterial regrowth.

The Time-kill kinetics were not monitored in the study. The checkerboard assay was not used for MBC determination, but for determination of drug synergy.

5. Why were these FIC interpretations used. The reference to this is another article that finally leads to <https://pubmed.ncbi.nlm.nih.gov/11168186/>. The interpretation here seems to be different. So can the authors explain this?

**The FICs were interpretated as.**

FICI ≤ 0.5 – synergism; 0.5 < FICI < 1– additive; 1 ≤ FICI < 2 – indifference; and FICI ≥ 2 – antagonism, which are in agreement with the cited article and your reference article

[https://pubmed.ncbi.nlm.nih.gov/11168186/](https://pubmed.ncbi.nlm.nih.gov/11168186/).

6. What were the clinical outcomes in treating these patients? What were the antibiotics used?

The requested information has been provided in **Table S1**

7. The language needs to be looked into. Eg Ceftalozane tazobactam is wrongly spelled.

The entire manuscript has been re-read and revised.
