# Supplementary material for: Synergistic antibacterial effects of colistin in combination with aminoglycoside, carbapenems, cephalosporins, fluoroquinolones, tetracyclines, fosfomycin, and piperacillin on multidrug resistant Klebsiella pneumoniae isolates
Source: PLoS One. 2021 Jan 6;16(1):e0244673. doi: 10.1371/journal.pone.0244673 (PMC7787437; doi:10.1371/journal.pone.0244673)
Supplement: Attachment — Submitted filename: Respose to reviewers.docx [file pone.0244673.s005.docx]

Additional Editor Comments

The authors need to clarify regarding the isolates and provide justification to call them "clinical isolates". Also treatment with antibiotics for colonisers should be justified.

Reviewer #2: Dear Authors

The answer to the reviewer explains that the isolates was for surveillance of the throat, rectum etc. But the manuscript still calls them clinical isolates.

Response: The isolates used for this study were obtained from patients receiving treatment in tertiary hospitals.

Furthermore, it is important to mention that colonizers can become opportunistic pathogens especially in immunocompromised individuals.

The clarity of the two is important in the fact that colonizers are not treated with antibiotics whereas pathogens are. Hence this distinction has to be made more distinctly.

Response: In response to your queries, we have replaced the term clinical isolates with *Klebsiella pneumonia* isolates. We also want to note that the antibiotics treatments were administered prior to inclusion into the surveillance study.

It is also important to mention Enterobacterales as the order rather than Enterobacteriaceae as a family is accepted nomenclature in the present times.

Corrected throughout the manuscript.
